# Potential Inhibitory Effect of Miltefosine against Terbinafine-Resistant *Trichophyton indotineae*

**DOI:** 10.3390/pathogens12040606

**Published:** 2023-04-17

**Authors:** Iman Haghani, Javad Akhtari, Zahra Yahyazadeh, Amirreza Espahbodi, Firoozeh Kermani, Javad Javidnia, Mohammad Taghi Hedayati, Tahereh Shokohi, Hamid Badali, Ali Rezaei-Matehkolaei, Seyed Reza Aghili, Ahmed Al-Rawahi, Ahmed Al-Harrasi, Mahdi Abastabar, Abdullah M. S. Al-Hatmi

**Affiliations:** 1 Invasive Fungi Research Center, Communicable Diseases Institute, Mazandaran University of Medical Sciences, Sari 48157-33971, Iran; 2 Department of Medical Mycology, School of Medicine, Mazandaran University of Medical Sciences, Sari 48157-33971, Iran; 3 Immunogenetics Research Center, Department of Medical Nanotechnology, School of Advanced Technologies in Medicine, Mazandaran University of Medical Sciences, Sari 48157-33971, Iran; 4 Student Research Committee, School of Medicine, Mazandaran University of Medical Sciences, Sari 48157-33971, Iran; 5 Department of Molecular Microbiology & Immunology, South Texas Center for Emerging Infectious Diseases, The University of Texas at San Antonio, San Antonio, TX 78249-0600, USA; 6 Department of Medical Mycology, School of Medicine, Infectious and Tropical Diseases Research Center, Health Research Institute, Ahvaz Jundishapur University of Medical Sciences, Ahvaz 61357-15794, Iran; 7 Natural & Medical Sciences Research Centre, University of Nizwa, Nizwa 616, Oman; 8 Center of Expertise in Mycology, Radboud University Medical Center/Canisius Wilhelmina Hospital, 6532 SZ Nijmegen, The Netherlands

**Keywords:** miltefosine, *T. mentagrophytes*, *T. interdigitale*, *T. indotineae*, terbinafine

## Abstract

Several prolonged and significant outbreaks of dermatophytosis caused by *Trichophyton indotineae*, a new emerging terbinafine-resistant species, have been ongoing in India in recent years, and have since spread to various countries outside Asia. Miltefosine, an alkylphosphocholine, is the most recently approved drug for the treatment of both visceral and cutaneous leishmaniasis. Miltefosine in vitro activity against terbinafine-resistant and susceptible *T. mentagrophytes*/*T. interdigitale* species complex, including *T. indotineae*, is limited. The current study aimed to assess miltefosine’s in vitro activity against dermatophyte isolates, which are the most common causes of dermatophytosis. Miltefosine, terbinafine, butenafine, tolnaftate, and itraconazole susceptibility testing was performed using Clinical and Laboratory Standards Institute broth microdilution methods (CLSI M38-A3) against 40 terbinafine-resistant *T. indotineae* isolates and 40 terbinafine-susceptible *T. mentagrophytes*/*T. interdigitale* species complex isolates. Miltefosine had MIC ranges of 0.063–0.5 µg/mL and 0.125–0.25 µg/mL against both terbinafine-resistant and susceptible isolates. In terbinafine-resistant isolates, the MIC_50_ and MIC_90_ were 0.125 µg/mL and 0.25 µg/mL, respectively, and 0.25 µg/mL in susceptible isolates. Miltefosine had statistically significant differences in MIC results when compared to other antifungal agents (*p*-value 0.05) in terbinafine-resistant strains. Accordingly, the findings suggest that miltefosine has a potential activity for treating infections caused by terbinafine-resistant *T. indotineae.* However, further studies are needed to determine how well this in vitro activity translates into in vivo efficacy.

## 1. Introduction

Dermatophytes are a group of keratinophilic fungi that commonly invade human and animal keratinized tissues and cause a variety of infections known as dermatophytosis [1,2,3]. According to the WHO, dermatophytosis affects 20–25% of the world’s population [4]. Dermatophytes are classified into seven genera, namely *Arthroderma*, *Epidermophyton*, *Lophophyton*, *Microsporum*, *Nannizzia*, *Paraphyton* and *Trichophyton*, according to the most recent taxonomy [5]. In contrast to the past, when *T. rubrum* was the most common *Trichophyton* species, there has been an unprecedented increase in the incidence of *T. mentagrophytes*/*interdigitale* species complex-associated dermatophytosis in recent years [6,7]. The main therapeutic strategies for dermatophytosis are systemic and topical treatment with allylamine and triazole drugs based on terbinafine and itraconazole. Terbinafine is the first-line treatment for *Trichophyton* species skin infections due to its stable clinical effect and low recurrence rate [8,9]. However, it was recently demonstrated that unsupervised and long-term use of ultra-potent topical corticosteroids, primarily clobetasol propionate, a class IV topical glucocorticoid, resulted in the occurrence of severe and extensive therapy-refractory tinea caused by *T. indotineae*, particularly in India [10,11]. Infections by this new emerging species are currently being reported from countries on all continents, with a significant percentage of reduced susceptibility or resistance to terbinafine due to excessive use of topical glucocorticoids [12,13,14,15,16,17,18,19,20,21,22,23,24]. A multicenter study in India found that over 76% of *T. indotineae* isolates were terbinafine resistant in vitro, and a significant number of patients infected with this emerging species no longer respond satisfactorily to topical or oral terbinafine treatment [23]. It’s worth noting that *T. indotineae* was previously known as *T. mentagrophytes* genotype VIII [25,26]. *Trichophyton indotineae*, on the other hand, is morphologically indistinguishable from *T. mentagrophytes*, has an anthropophilic rather than zoophilic transmission pattern, and has a high level of terbinafine resistance [16,27]. *Trichophyton indotineae* dermatophytosis appears to be widely transmitted from person to person. It frequently begins as difficult-to-treat tinea corporis, tinea cruris, tinea genitalis, or tinea faciei, with inflammatory or hyper-pigmented scaly and severely itchy lesions occurring concurrently. Although the lesions are difficult to diagnose as dermatophytosis most of the time, the lesions in the groin eventually spread posteriorly to the gluteal region and the trunk and extremities as a direct spread, resulting in large lesions [28].

As previously stated, significant numbers of *T. indotineae* infections have not responded to terbinafine topical and oral treatment [29,30,31]. This corresponds to the occurrence of one or more point mutations with subsequent amino acid substitutions at the squalene epoxidase (*SQLE*) gene positions L393F or F397L [32]. Given the aforementioned issue, itraconazole was proposed as a substitute choice strategy for treating this pathogen’s dermatophytosis [29,33]. While a recent study found conflicting results in reducing the effect of this agent on *T. indotineae* due to the *SQLE* gene point mutation c.1342G>A [34]. These findings suggest that the antifungal arsenal for *T. indotineae* infections resistant to terbinafine treatment is limited, emphasizing the need for ongoing research into novel and effective options for controlling such recalcitrant pathogens.

Miltefosine, an alkylphosphocholine agent, was originally developed as an anticancer drug in the 1980s, but it is now the only FDA-approved antiparasitic medication for the treatment of cutaneous, mucosal, and visceral leishmaniasis [35]. Furthermore, the CDC recommended agent as first-line therapy for infections caused by free-living amoebas [36]. Miltefosine has also shown promising in vitro activity against a variety of clinically significant molds and yeasts, including dimorphic fungi, *Aspergillus* spp., *Candida* spp., *Cryptococcus* spp., *Fusarium* spp., *Rhizopus* spp., and *Scedosporium* spp. [37,38,39,40,41,42,43,44]. However, there is little information about this compound’s antifungal activity against dermatophytes, and its activity against *T. indotineae* is unknown. As a result, the current study aimed to: (i) evaluate miltefosine’s in vitro antifungal activity against a large collection of clinical terbinafine resistance *T. indotineae* and 40 susceptible *T. mentagrophytes*/*interdigitale* species complex isolates; and (ii) compare miltefosine’s activity against these groups of *Trichophyton* species with five commercially available topical and systemic antifungal drugs.

## 2. Materials and Methods

### 2.1. Sample Collection

Clinical isolates of dermatophytes, 40 terbinafine-resistant *T. indotineae* (MIC ≥ 4 µg/mL) and 40 susceptible *T. mentagrophytes*/*interdigitale* species complex isolates (MIC ≤ 2 µg/mL) from the Mazandaran University of Medical Sciences collection in Sari, Iran, were included.

### 2.2. Molecular Identification

All isolates had previously been confirmed to the species level using a combination of phenotypic characteristics and DNA sequence analysis of the internal transcribed space (ITS rDNA) [45,46]. To determine whether a mutation in the *SQLE* gene was responsible for the high levels of terbinafine MIC in terbinafine-resistant isolates, the partial *SQLE* gene was amplified and sequenced in terbinafine-resistant *T. indotineae* strains using the specific primers TrSQLE-F1 (5′-ATGGTTGTAGAGGCTCCTCCC-3′) and TrSQLE-R1 (5′-CTAGCTTTGAAGTTC [47]. The sequences were deposited in GenBank under the accession numbers OQ214837-OQ214877. Missense mutations corresponding to the Phe397Leu substituted amino acid in the SQLE protein were found in 32 terbinafine-resistant isolates.

### 2.3. Antifungal Susceptibility Testing

Antifungal susceptibility testing was carried out using broth microdilution methods described in the Clinical and Laboratory Standards Institute (CLSI) M38-A3 document, using RPMI 1640 medium (Sigma Chemical Co.) buffered to pH 7.0 with 0.165 M-morpholinepropanesulfonic acid (MOPS) (Sigma) with L-glutamine and no bicarbonate [48]. Miltefosine (Cayman Chemical, Ann Arbor, MI, USA), itraconazole (Janssen Pharmaceutica, Beerse, Belgium), ketoconazole, terbinafine, butenafine, and tolnaftate (Sigma-Aldrich, St. Louis, MO, USA) stock solutions were prepared in DMSO (dimethylsulfoxide) at the concentrations ranging from 0.016 to 16 µg/mL. Briefly, conidial suspensions were prepared by scraping the surface of mature colonies (two weeks old) with a sterile cotton swab moistened with a sterile physiological saline solution containing 0.05% tween 20–40 and spectrophotometrically adjusting to optical densities (ODs) ranging from 65% to 70% transmission at a 530 nm wavelength. Inoculum suspensions were diluted 1:50 in RPMI 1640 medium, with the final inoculum in assay wells ranging from 1 × 10^3^ to 3 × 10^3^ CFU/mL. After 72 h of incubating the microdilution plates at 35 °C, the results were visually read. The MIC was visually determined as the lowest drug concentration that inhibited fungal growth by 80% or more. As quality control strains, *Candida parapsilosis* (ATCC 22019), *Candida krusei* (ATCC 6258), and *Aspergillus flavus* (ATCC 2004304) were used, and all antifungal susceptibility tests were performed in duplicate.

### 2.4. Statistical Analysis

The statistical SPSS package version 20 and GraphPad Prism version 7 were used to calculate Kruskal-Wallis differences in mean values. *p*-values of 0.05 were considered statistically significant.

## 3. Results

Table 1 shows the antifungal susceptibility profiles of miltefosine and five comparator drugs, including ketoconazole, terbinafine, itraconazole, butenafine, and tolnaftate, against tested *Trichophyton* species. Miltefosine’s MICs against terbinafine-resistant *T. indotineae* isolates ranged from 0.063 to 0.5 µg/mL, compared to 4 to 4 µg/mL for terbinafine and butenafine, 0.016 to 16 µg/mL for tolnaftate and itraconazole, and 0.032 to 1 µg/mL for ketoconazole. Miltefosine with Geometric Mean (GM) MIC 0.15 µg/mL and itraconazole with GM MIC 0.14 µg/mL exhibited potent activity against terbinafine-resistant *T. indotineae*, followed by ketoconazole (GM MIC 0.26 µg/mL), tolnaftate (GM MIC 7.34 µg/mL), terbinafine (GM MIC 4 µg/mL), and butenafine (GM MIC 4 µg/mL), respectively.

In terms of MIC_50_, miltefosine and itraconazole had the same activity (0.125 µg/mL) against terbinafine-resistant *T. indotineae* followed by ketoconazole (0.25 µg/mL), butenafine (4 µg/mL), terbinafine (4 µg/mL) and tolnaftate (16 µg/mL). In comparison to ketoconazole (0.5 µg/mL), itraconazole (1 µg/mL), terbinafine (4 µg/mL), butenafine (4 µg/mL), and tolnaftate (16 µg/mL), miltefosine had the lowest MIC_90_ value (0.25 µg/mL) against terbinafine-resistant *T. indotineae* isolates. Except for miltefosine (*p* = 0.075), the difference in mean MIC values between terbinafine-resistant and terbinafine-susceptible isolates was significant for all drugs (*p =* 0.001) (Table 2).

Miltefosine had a significantly lower GM MIC value (0.172 µg/mL) in the terbinafine-resistant group than ketoconazole, terbinafine, butenafine, and tolnaftate (0.323, 4, 4, and 12.925 µg/mL, respectively) (*p* = 0.001), but this difference was not significant with itraconazole (1.415 µg/mL) (*p* = 0.448) (Table 3).

The difference in the GM MIC values of itraconazole and ketoconazole in the terbinafine-resistant group was not statistically significant (*p* = 0.589); both had significantly lower mean MIC values compared to terbinafine, butenafine, and tolnaftate (*p* = 0.001). Furthermore, the GM MICs of butenafine and terbinafine were lower than those of tolnaftate (*p* = 0.001). While itraconazole had a lower GM MIC value (0.061 µg/mL) in the terbinafine-susceptible group compared to miltefosine, ketoconazole, and tolnaftate (0.193, 0.165, and 0.195 µg/mL, respectively) (*p* = 0.001), other comparisons were not significant (*p* > 0.05) (Table 3).

## 4. Discussion

A regular search for new potential antifungal compounds is an obvious strategy for combating antifungal resistance, and in this study, we highlighted miltefosine’s in vitro anti-dermatophyte activity against a collection of terbinafine-resistant and terbinafine-susceptible *Trichophyton* clinical isolates. *Trichophyton indotineae*, a new dermatophyte species capable of causing dermatophytosis resistant to terbinafine treatment, has emerged globally in recent years [29,31]. Itraconazole was introduced as a substituted choice in cases where terbinafine treatment failed [29,31,42], but a recent German case series report on *T. indotineae* with reduced susceptibility to itraconazole [34] has maintained the challenge of difficult-to-treat *T. indotineae*. Although no reliable breakpoints have been defined for itraconazole, based on the CLSI protocol, *T. mentagrophytes* isolates with MIC values of 0.25 to 0.5 μg/mL might be considered resistant isolates [4]. According to our findings (Table 1), the MIC values of terbinafine-resistant isolates for itraconazole (MIC_90_ = 1 μg/mL) compared to miltefosine (MIC_90_ = 0.25 μg/mL) was higher and additionally, there was no significant difference between the GM MIC values of two antifungals in the terbinafine-resistance group which imply the application in favor of miltefosine instead of itraconazole.

Few studies have been conducted on miltefosine’s antifungal activity against pathogenic fungi. Miltefosine is effective in vitro and in vivo against a limited number of molds, dimorphic fungi, and yeasts [38,40,42,44], but its potential activity against terbinafine-resistant *Trichophyton* strains has yet to be investigated. Miltefosine and itraconazole were more potent against *T. indotineae* isolates with high MICs for terbinafine in the current study, and miltefosine had lower MIC_50_, MIC_90_, and MIC range values against terbinafine-resistant isolates when compared to others.

The potency of luliconazole, a new topical imidazole, against zoophilic and anthropophilic *Trichophyton* isolates has recently been studied [3,49]. However, this antifungal medication has not been marketed in many countries, was developed in a 10% solution for the topical treatment of nail infections, and research on its efficacy in the treatment of recalcitrant and extensive tinea corporis and tinea corporis is limited. A recent randomized pragmatic trial in India found that fluconazole, griseofulvin, itraconazole, and terbinafine had limited efficacy in the treatment of chronic relapsing tinea corporis and tinea cruris [33]. Finally, ketoconazole, butenafine, and tolnaftate had higher GM MIC values (4, 4, and 0.5 μg/mL, respectively) than miltefosine to inhibit terbinafine-resistant *T. indotineae* isolates [50]. Unfortunately, there is no in vitro or in vivo data on the efficacy of these topical agents against terbinafine-resistant dermatophytes, and it appears that topical medications have the least effect in the global management of recalcitrant dermatophytosis caused by *T. indotineae*. Miltefosine is currently prescribed orally as the most effective and safe drug with the potential to treat all major clinical presentations of leishmaniasis, though topical formulations for the treatment of cutaneous leishmaniasis appear ineffective [51]. Miltefosine’s in vitro potent activity against a variety of medically important yeasts and molds has been validated in the context of fungal infection [37,38,39,40,41,42,43]. However, the US Company Profounda, Inc. announced in November 2021 that it had received FDA orphan drug designation approval for using miltefosine to treat invasive candidiasis [52]. Unlike itraconazole and terbinafine, which are both oral treatments, miltefosine can be applied topically to treat this infection. Given its lower side effects than miltefosine and its different mechanisms of action on the fungal cell membrane [40], itraconazole may be a promising candidate for therapeutic strategies. Daily administration of miltefosine for the treatment of leishmaniasis (2.5 mg/kg of body weight/day for 28 days) resulted in a mean maximum concentration of the drug in serum at day 23 of treatment of 70 µg/mL. Furthermore, on days 26 to 28 of treatment, the median minimum concentration of the drug in serum for children who received 2.5 mg/kg of body weight/day for 28 days was 26 μg/mL. According to the findings of this study, it is possible that this dose of miltefosine will achieve the desired serum level for antifungal activity [53]. Given these considerations, miltefosine may be considered a potential therapeutic alternative for managing dermatophytosis cases caused by *Trichophyton* species that are resistant or have reduced susceptibility to terbinafine, itraconazole, and other common antifungal agents.

## 5. Conclusions

There are few other options for treating terbinafine-resistant dermatophytosis, our findings are promising and suggest that miltefosine may be useful in treating infections caused by terbinafine-resistant *T. mentagrophytes*/*T. interdigitale* species complex isolates. However, more research is needed to determine how in vitro activity translates into clinical outcomes for the treatment of dermatophytosis.

## Figures and Tables

**Table 1:** In vitro susceptibility of 80 terbinafine-susceptible and resistant *Trichophyton* isolates to Miltefosine and five routine antifungal agents.

Antifungal Drug	Group	MIC (μg/ML)				
		Range	MIC_50_	MIC_90_	GM	Mode
Itraconazole	Terbinafine-resistant	0.016–16	0.125	1	0.140	0.063
	Terbinafine-susceptible	0.016–0.25	0.063	0.125	0.048	0.032
	Total	0.016–16	0.063	0.25	0.082	0.063
Terbinafine	Terbinafine-resistant	4–>4	4	4	4.000	4
	Terbinafine-susceptible	0.004–2	0.032	0.25	0.045	0.032
	Total	0.004–<4	2	4	0.423	4
Butenafine	Terbinafine-resistant	4–<4	4	4	4.000	4
	Terbinafine-susceptible	0.004–2	0.125	0.25	0.086	0.125
	Total	0.004–<4	2	4	0.586	4
Tolnaftate	Terbinafine-resistant	0.016–<16	16	16	7.340	16
	Terbinafine-susceptible	0.032–0.5	0.125	0.5	0.139	0.25
	Total	0.016–<16	0.5	16	1.011	16
Ketoconazole	Terbinafine-resistant	0.032–1	0.25	0.5	0.264	0.25
	Terbinafine-susceptible	0.125–0.25	0.125	0.25	0.157	0.125
	Total	0.032–1	0.25	0.5	0.203	0.125
Miltefosine	Terbinafine-resistant	0.063–0.5	0.125	0.25	0.154	0.125
	Terbinafine-susceptible	0.125–0.25	0.25	0.25	0.183	0.25
	Total	0.063–0.5	0.125	0.25	0.168	0.125

**Table 2:** Comparison of each drug’s mean MIC value between two groups.

Antifungal Drug	Group	MIC Means (Std. Deviation)	*p* Value
Itraconazole	Terbinafine-resistant	1.415 (4.252)	0.001
	Terbinafine-susceptible	0.061 (0.047)	
Terbinafine	Terbinafine-resistant	4.000 (0.000)	<0.0001
	Terbinafine-susceptible	0.147 (0.330)	
Butenafine	Terbinafine-resistant	4.000 (0.000)	<0.0001
	Terbinafine-susceptible	0.169 (0.317)	
Tolnaftate	Terbinafine-resistant	12.925 (6.234)	<0.0001
	Terbinafine-susceptible	0.196 (0.152)	
Ketoconazole	Terbinafine-resistant	0.323 (0.192)	<0.0001
	Terbinafine-susceptible	0.166 (0.059)	
Miltefosine	Terbinafine-resistant	0.172 (0.086)	0.075
	Terbinafine-susceptible	0.194 (0.063)	

**Table 3:** Drugs mean MIC comparison in each group.

Antifungal Drug		Terbinafine-Resistant Group			Terbinafine-Susceptible Group		
		Mean Difference	Std. Error	Sig.	Mean Difference	Std. Error	Sig.
Miltefosine	Itraconazole	−1.243	0.672	0.448	0.132	0.012	<0.0001
Miltefosine	Ketoconazole	−0.151	0.033	<0.0001	0.028	0.014	0.321
Miltefosine	Tolnaftate	−12.753	0.986	<0.0001	−0.002	0.026	1.000
Miltefosine	Butenafine	−3.828	0.014	<0.0001	0.025	0.051	0.996
Miltefosine	Terbinafine	−3.828	0.014	<0.0001	0.047	0.053	0.948
Itraconazole	Ketoconazole	1.092	0.673	0.589	−0.104	0.012	<0.0001
Itraconazole	Tolnaftate	−11.510	1.193	<0.0001	−0.134	0.025	<0.0001
Itraconazole	Butenafine	−2.585	0.672	0.005	−0.107	0.051	0.303
Itraconazole	Terbinafine	−2.585	0.672	0.005	−0.085	0.053	0.592
Ketoconazole	Tolnaftate	−12.603	0.986	<0.0001	−0.030	0.026	0.848
Ketoconazole	Butenafine	−3.677	0.030	<0.0001	−0.003	0.051	1.000
Ketoconazole	Terbinafine	−3.677	0.030	<0.0001	0.019	0.053	0.999
Tolnaftate	Butenafine	8.925	0.986	<0.0001	0.027	0.056	0.996
Tolnaftate	Terbinafine	8.925	0.986	<0.0001	0.049	0.057	0.955
Butenafine	Terbinafine	0.0001	0.0001	1.000	0.022	0.072	1.000

## Data Availability

Data are available in Table 1, Table 2 and Table 3.

## References

[1] Dabas Y., Xess I., Singh G., Pandey M., Meena S. (2017). Molecular Identification and Antifungal Susceptibility Patterns of Clinical Dermatophytes Following CLSI and EUCAST Guidelines. J. Fungi.

[2] Salehi Z., Shams-Ghahfarokhi M., Razzaghi-Abyaneh M. (2021). Molecular Epidemiology, Genetic Diversity, and Antifungal Susceptibility of Major Pathogenic Dermatophytes Isolated from Human Dermatophytosis. Front. Microbiol..

[3] Zareshahrabadi Z., Totonchi A., Rezaei-Matehkolaei A., Ilkit M., Ghahartars M., Arastehfar A., Motamedi M., Nouraei H., Lari M.S., Mohammadi T. (2021). Molecular identification and antifungal susceptibility among clinical isolates of dermatophytes in Shiraz, Iran (2017–2019). Mycoses.

[4] Ebert A., Monod M., Salamin K., Burmester A., Uhrlaß S., Wiegand C., Hipler U.-C., Krüger C., Koch D., Wittig F. (2020). Alarming India-wide phenomenon of antifungal resistance in dermatophytes: A multicentre study. Mycoses.

[5] De Hoog G.S., Dukik K., Monod M., Packeu A., Stubbe D., Hendrickx M., Kupsch C., Stielow J.B., Freeke J., Göker M. (2017). Toward a novel multilocus phylogenetic taxonomy for the dermatophytes. Mycopathologia.

[6] Rudramurthy S.M., Shankarnarayan S.A., Dogra S., Shaw D., Mushtaq K., Paul R.A., Narang T., Chakrabarti A. (2018). Mutation in the Squalene Epoxidase Gene of Trichophyton interdigitale and Trichophyton rubrum Associated with Allylamine Resistance. Antimicrob. Agents Chemother..

[7] Nenoff P., Verma S.B., Vasani R., Burmester A., Hipler U., Wittig F., Krüger C., Nenoff K., Wiegand C., Saraswat A. (2019). The current Indian epidemic of superficial dermatophytosis due to*Trichophyton mentagrophytes*—A molecular study. Mycoses.

[8] Haugh M., Helou S., Boissel J., Cribier B. (2002). Terbinafine in fungal infections of the nails: A meta-analysis of randomized clinical trials. Br. J. Dermatol..

[9] Niimi M., Firth N.A., Cannon R.D. (2010). Antifungal drug resistance of oral fungi. Odontology.

[10] Verma S., Madhu R. (2017). The Great Indian Epidemic of Superficial Dermatophytosis: An Appraisal. Indian J. Dermatol..

[11] Bishnoi A., Vinay K., Dogra S. (2018). Emergence of recalcitrant dermatophytosis in India. Lancet Infect. Dis..

[12] Nenoff P., Verma S.B., Ebert A., Süß A., Fischer E., Auerswald E., Dessoi S., Hofmann W., Schmidt S., Neubert K. (2020). Spread of Terbinafine-Resistant *Trichophyton mentagrophytes* Type VIII (India) in Germany—“The Tip of the Iceberg?”. J. Fungi.

[13] Jabet A., Brun S., Normand A.-C., Imbert S., Akhoundi M., Dannaoui E., Audiffred L., Chasset F., Izri A., Laroche L. (2022). Extensive Dermatophytosis Caused by Terbinafine-Resistant *Trichophyton indotineae*, France. Emerg. Infect. Dis..

[14] Dellière S., Joannard B., Benderdouche M., Mingui A., Gits-Muselli M., Hamane S., Alanio A., Petit A., Gabison G., Bagot M. (2022). Emergence of Difficult-to-Treat Tinea Corporis Caused by *Trichophyton mentagrophytes* Complex Isolates, Paris, France. Emerg. Infect. Dis..

[15] Sacheli R., Hayette M.-P. (2021). Antifungal Resistance in Dermatophytes: Genetic Considerations, Clinical Presentations and Alternative Therapies. J. Fungi.

[16] Klinger M., Theiler M., Bosshard P. (2021). Epidemiological and clinical aspects of *Trichophyton mentagrophytes/Trichophyton interdigitale* infections in the Zurich area: A retrospective study using genotyping. J. Eur. Acad. Dermatol. Venereol..

[17] Siopi M., Efstathiou I., Theodoropoulos K., Pournaras S., Meletiadis J. (2021). Molecular epidemiology and antifungal susceptibility of trichophyton isolates in greece: Emergence of terbinafine-resistant *Trichophyton mentagrophytes* type viii locally and globally. J. Fungi.

[18] Kong X., Tang C., Singh A., Ahmed S.A., Al-Hatmi A.M.S., Chowdhary A., Nenoff P., Gräser Y., Hainsworth S., Zhan P. (2021). Antifungal Susceptibility and Mutations in the Squalene Epoxidase Gene in Dermatophytes of the *Trichophyton mentagrophytes* Species Complex. Antimicrob. Agents Chemother..

[19] Posso-De Los Rios C.J., Tadros E., Summerbell R.C., Scott J.A. (2022). Terbinafine resistant Trichophyton indotineae isolated in patients with superficial dermatophyte infection in Canadian patients. J. Cutan. Med. Surg..

[20] Ngo T.M.C., Nu P.A.T., Le C.C., Ha T.N.T., Do T.B.T., Thi G.T. (2022). First detection of Trichophyton indotineae causing tinea corporis in Central Vietnam. Med. Mycol. Case Rep..

[21] Astvad K.M.T., Hare R.K., Jørgensen K.M., Saunte D.M.L., Thomsen P.K., Arendrup M.C. (2022). Increasing terbinafine resistance in Danish Trichophyton isolates 2019–2020. J. Fungi.

[22] Fattahi A., Shirvani F., Ayatollahi A., Rezaei-Matehkolaei A., Badali H., Lotfali E., Ghasemi R., Pourpak Z., Firooz A. (2021). Multidrug-resistant *Trichophyton mentagrophytes* genotype VIII in an Iranian family with generalized dermatophytosis: Report of four cases and review of literature. Int. J. Dermatol..

[23] Uhrlaß S., Verma S.B., Gräser Y., Rezaei-Matehkolaei A., Hatami M., Schaller M., Nenoff P. (2022). *Trichophyton indotineae*—An Emerging Pathogen Causing Recalcitrant Dermatophytoses in India and Worldwide—A Multidimensional Perspective. J. Fungi.

[24] Vineetha M., Sheeja S., Celine M., Sadeep M., Palackal S., Shanimole P., Saranya Das S. (2019). Profile of dermatophytosis in a tertiary care center in Kerala, India. Indian J. Dermatol..

[25] Verma S.B., Panda S., Nenoff P., Singal A., Rudramurthy S.M., Uhrlass S., Das A., Bisherwal K., Shaw D., Vasani R. (2021). The unprecedented epidemic-like scenario of dermatophytosis in India: II. Diagnostic methods and taxonomical aspects. Indian J. Dermatol. Venereol. Leprol..

[26] Kano R., Kimura U., Kakurai M., Hiruma J., Kamata H., Suga Y., Harada K. (2020). Trichophyton indotineae sp. nov.: A New Highly Terbinafine-Resistant Anthropophilic Dermatophyte Species. Mycopathologia.

[27] Saunte D., Pereiro-Ferreirós M., Rodríguez-Cerdeira C., Sergeev A., Arabatzis M., Prohić A., Piraccini B.M., Lecerf P., Nenoff P., Kotrekhova L.P. (2021). Emerging antifungal treatment failure of dermatophytosis in Europe: Take care or it may become endemic. J. Eur. Acad. Dermatol. Venereol..

[28] Khurana A., Gupta A., Sardana K., Sethia K., Panesar S., Aggarwal A., Ghadlinge M. (2020). A prospective study on patterns of topical steroids self-use in dermatophytoses and determinants predictive of cutaneous side effects. Dermatol. Ther..

[29] Verma S.B., Panda S., Nenoff P., Singal A., Rudramurthy S.M., Uhrlass S., Das A., Bisherwal K., Shaw D., Vasani R. (2021). The unprecedented epidemic-like scenario of dermatophytosis in India: I. Epidemiology, risk factors and clinical features. Indian J. Dermatol. Venereol. Leprol..

[30] Shen J.J., Arendrup M.C., Verma S., Saunte D.M.L. (2022). The emerging terbinafine-resistant Trichophyton epidemic: What Is the role of antifungal susceptibility testing?. Dermatology.

[31] Verma S.B., Panda S., Nenoff P., Singal A., Rudramurthy S.M., Uhrlass S., Das A., Bisherwal K., Shaw D., Vasani R. (2021). The unprecedented epidemic-like scenario of dermatophytosis in India: III. Antifungal resistance and treatment options. Indian J. Dermatol. Venereol. Leprol..

[32] Burmester A., Hipler U.-C., Hensche R., Elsner P., Wiegand C. (2019). Point mutations in the squalene epoxidase gene of Indian ITS genotype VIII *T. mentagrophytes* identified after DNA isolation from infected scales. Med. Mycol. Case Rep..

[33] Singh S., Chandra U., Anchan V., Verma P., Tilak R. (2020). Limited effectiveness of four oral antifungal drugs (fluconazole, griseofulvin, itraconazole and terbinafine) in the current epidemic of altered dermatophytosis in India: Results of a randomized pragmatic trial. Br. J. Dermatol..

[34] Brasch J., Gräser Y., Beck-Jendroscheck V., Voss K., Torz K., Walther G., Schwarz T. (2021). “Indian” strains of *Trichophyton mentagrophytes* with reduced itraconazole susceptibility in Germany. JDDG J. Dtsch. Dermatol. Ges..

[35] Dorlo T.P.C., Balasegaram M., Beijnen J.H., De Vries P.J. (2012). Miltefosine: A review of its pharmacology and therapeutic efficacy in the treatment of leishmaniasis. J. Antimicrob. Chemother..

[36] Alli A., Ortiz J.F., Cox Á.M., Armas M., Orellana V.A. (2021). Miltefosine: A Miracle Drug for Meningoencephalitis Caused by Free-Living Amoebas. Cureus.

[37] Vila T., Ishida K., Seabra S.H., Rozental S. (2016). Miltefosine inhibits Candida albicans and non-albicans Candida spp. biofilms and impairs the dispersion of infectious cells. Int. J. Antimicrob. Agents.

[38] Imbert S., Palous M., Meyer I., Dannaoui E., Mazier D., Datry A., Fekkar A. (2014). In Vitro Combination of Voriconazole and Miltefosine against Clinically Relevant Molds. Antimicrob. Agents Chemother..

[39] Rossi D.C.P., Spadari C.D.C., Nosanchuk J.D., Taborda C.P., Ishida K. (2017). Miltefosine is fungicidal to Paracoccidioides spp. yeast cells but subinhibitory concentrations induce melanisation. Int. J. Antimicrob. Agents.

[40] Biswas C., Sorrell T., Djordjevic J., Zuo X., Jolliffe K., Chen S.C.-A. (2013). In vitro activity of miltefosine as a single agent and in combination with voriconazole or posaconazole against uncommon filamentous fungal pathogens. J. Antimicrob. Chemother..

[41] Vila T., Quintanilha N.S., Rozental S. (2015). Miltefosine is effective against Candida albicans and Fusarium oxysporum nail biofilms in vitro. J. Med. Microbiol..

[42] Rollin-Pinheiro R., Almeida Y.D.C., Rochetti V.P., Xisto M.I.D.D.S., Borba-Santos L.P., Rozental S., Barreto-Bergter E. (2021). Miltefosine against Scedosporium and Lomentospora Species: Antifungal Activity and Its Effects on Fungal Cells. Front. Cell. Infect. Microbiol..

[43] Nosratabadi M., Akhtari J., Faeli L., Haghani I., Aghili S.R., Shokohi T., Hedayati M.T., Zarrinfar H., Mohammadi R., Najafzadeh M.J. (2022). In Vitro Antifungal Susceptibility Profile of Miltefosine against a Collection of Azole and Echinocandins Resistant *Fusarium* Strains. J. Fungi.

[44] Haghani I., Yahyazadeh Z., Hedayati M.T., Shokohi T., Badali H., Khojasteh S., Akhtari J., Javidnia J., Moazeni M., Al-Harrasi A. (2023). Antifungal activity of miltefosine against both azole-susceptible and resistant Aspergillus strains. Int. J. Antimicrob. Agents.

[45] Abastabar M., Jedi A., Guillot J., Ilkit M., Eidi S., Hedayati M.T., Shokohi T., Ghazvini R.D., Rezaei-Matehkolaei A., Katiraee F. (2019). In vitro activities of 15 antifungal drugs against a large collection of clinical isolates of *Microsporum canis*. Mycoses.

[46] Haghani I., Shams-Ghahfarokhi M., Asl A.D., Shokohi T., Hedayati M.T. (2019). Molecular identification and antifungal susceptibility of clinical fungal isolates from onychomycosis (uncommon and emerging species). Mycoses.

[47] Yamada T., Maeda M., Alshahni M.M., Tanaka R., Yaguchi T., Bontems O., Salamin K., Fratti M., Monod M. (2017). Terbinafine Resistance of Trichophyton Clinical Isolates Caused by Specific Point Mutations in the Squalene Epoxidase Gene. Antimicrob. Agents Chemother..

[48] Wayne P. (2017). Reference Method for Broth Dilution Antifungal Susceptibility Testing of Filamentous Fungi.

[49] Shaw D., Singh S., Dogra S., Jayaraman J., Bhat R., Panda S., Chakrabarti A., Anjum N., Chowdappa A., Nagamoti M. (2020). MIC and Upper Limit of Wild-Type Distribution for 13 Antifungal Agents against a *Trichophyton mentagrophytes*-*Trichophyton interdigitale* Complex of Indian Origin. Antimicrob. Agents Chemother..

[50] Brennan B., Leyden J.J. (1997). Overview of topical therapy for common superficial fungal infections and the role of new topical agents. J. Am. Acad. Dermatol..

[51] Van Bocxlaer K., Yardley V., Murdan S., Croft S.L. (2016). Topical formulations of miltefosine for cutaneous leishmaniasis in a BALB/c mouse model. J. Pharm. Pharmacol..

[52] https://www.prnewswire.com/news-releases/fda-grants-profounda-orphan-drug-designation-approval-for-treatment-of-treatment-of-invasive-candidiasis-with-miltefosine-301417861.html.

[53] Dorlo T.P.C., van Thiel P.P.A.M., Huitema A.D.R., Keizer R.J., de Vries H.J.C., Beijnen J.H., de Vries P.J. (2008). Pharmacokinetics of Miltefosine in Old World Cutaneous Leishmaniasis Patients. Antimicrob. Agents Chemother..

